# Theory of the Production of Males and Females

**Published:** 1850-09

**Authors:** Silas Hubbard


### *Theory of the Production of Males and Females.*

By Silas Hubbard, M. D.

To the Editor of the Buffalo Medical Journal.

Sir,—Many have been the theories of generation which have been either proved to be, or are now regarded as errroneous, and are merely mentioned matters of history. Among these by-gones, are all the ancient theories of the causes of the production of males or females: but as this subject still occupies the serious attention of very many respectable physicians, I may be excused for offering the following new and original theory, viz.: that males are begotten from one to ten days before, and females from one to ten days after the courses of the mother. In proof of this observation, I shall now merely say, that it has invariably held true in all the cases I have had the means of knowing, which are half a dozen.

August 10th, 1850.

